# Drug Consumption and Hydration Status: Analysis of the Associations in an Elder Population

**DOI:** 10.3390/nu16162632

**Published:** 2024-08-09

**Authors:** Sara López Oliva, Carmen Morais-Moreno, Alejandra Carretero-Krug, María de Lourdes Samaniego-Vaesken, Ana M. López-Sobaler, Teresa Partearroyo, Ana M. Puga

**Affiliations:** 1 Grupo USP-CEU de Excelencia “Nutrición para la Vida (Nutrition for Life)”, Ref: E02/0720, Departamento de Ciencias Farmacéuticas y de la Salud, Facultad de Farmacia, Universidad San Pablo-CEU, CEU Universities, Urbanización Montepríncipe, 28660 Boadilla del Monte, Spain; sara.lopezoliva@ceu.es (S.L.O.); carmen.moraismoreno@ceu.es (C.M.-M.); alejandra.carreterokrug@ceu.es (A.C.-K.); l.samaniego@ceu.es (M.d.L.S.-V.); t.partearroyo@ceu.es (T.P.); 2 Instituto CEU Alimentación y Sociedad, Facultad de Farmacia, Universidad San Pablo-CEU, CEU Universities, Urbanización Montepríncipe, 28660 Boadilla del Monte, Spain; 3 Grupo de Investigación VALORNUT, Departamento de Nutrición y Ciencia de los Alimentos, Facultad de Farmacia, Universidad Complutense de Madrid, 28040 Madrid, Spain; asobaler@ucm.es; 4 Instituto de Investigaciones Sanitarias San Carlos (IdISSC), 28040 Madrid, Spain

**Keywords:** drug consumption, hydration status, dehydration, elderly, polypharmacy, total body water, diuretics, cardiovascular drugs, genito-urinary drugs, water intake

## Abstract

Hydration status plays a key role in healthy ageing, and it is potentially affected by several factors, including drug consumption. However, research on this issue to date is scarce, especially in highly vulnerable groups, such as the elderly. We aimed to study the relationship linking hydration status, analysed by means of a validated questionnaire, 24 h urine analysis, body composition assessment, and drug consumption in a sample of old adults. A total of 144 elders were included in the study. Cardiovascular drug consumption was significantly associated with a lower water intake in men (*β* = −0.282, *p* = 0.029). Moreover, urinary analysis revealed that total drug intake as well as the consumption of diuretics and cardiovascular drugs were associated with poorer hydration status, whereas genito-urinary drugs were associated with an opposite effect, and these results were confirmed in terms of body composition. Hence, total drug consumption (*β* = −0.205), diuretic (*β* = −0.408), cardiovascular (*β* = −0.297), and genito-urinary drugs (*β* = 0.298) were significantly associated (*p* < 0.05) with total body water. The obtained results confirmed the impact of chronic treatment with certain drugs on hydration status. Nutritional interventions may be of great interest in certain population groups in order to prevent complications due to altered hydration status.

## 1. Introduction

Ensuring healthy and quality ageing is one of the main concerns of the 21st century, especially considering the increasing population, particularly in countries with higher life expectancy. For example, in the European Union, between 2001 and 2020, the proportion of the population aged 65 and over increased from 16 to 21% [1]. Particularly, in Spain in 2023, 20.15% of the population was aged 65 or older [2]. Current available evidence suggests that healthy nutrition, avoiding nutritional or dietary deficiencies, is more important for healthy ageing than is generally perceived [3]. In fact, nutrition prescription strategies in this population group, and also in the pre-elderly years, seem to be of interest to mitigate the consequences of the epidemic of unhealthy ageing before they become irreversible [4].

Water is one of the most important and essential nutrients, accounting for approximately 60% of the body mass of an adult [5] and varying according to sex: water typically comprises 50–55% of a female’s body weight, as opposed to 60–70% in men [6]. However, this percentage declines with age [7]. It plays a key role in thermoregulation, blood pressure maintenance, biochemical pathways, as well as nutrient transport and waste removal [7]. Dehydration is the clinical term generally used to refer to a deficiency in total body water [8]. The elderly are highly vulnerable to suffer from dehydration with the subsequent associated consequences for both health and life quality. In fact, it acts as an independent factor for hospital length of stay, readmission, intensive care, in-hospital mortality, and poor prognosis [9,10]. It was estimated that the prevalence of dehydration in community-dwelling older adults ranged from 1 to 60% [11]. Among long-term care residents and hospital inpatients, dehydration prevalences of 20.0 to 38.3% and around 37.0%, respectively, have been calculated [10,12,13], with differences between sexes [12]. In any case, it should be recognised that dehydration assessment is a major clinical challenge due to its complexity, variable pathophysiology, non-specific clinical symptoms, as well as lack of international consensus on definition and diagnosis [8].

In the last few years, there has been a marked increase in drug consumption, especially among the elderly from developed countries. According to the World Health Organisation (WHO), polypharmacy is the concurrent use of multiple drugs, including over-the-counter, prescription, and/or traditional and complementary medicines [14]. In fact, the term polypharmacy has been recently introduced as the chronic consumption of five or more drugs [15,16]. It has been estimated that around 44.2–57.7% of adults older than 65 years are chronic consumers of five or more drugs, and additionally, 9.1–23.2% chronically use ten or more drugs [17,18,19,20]. Despite polypharmacy in the elderly being frequently needed for the management of chronic diseases, it is associated with an increased risk of adverse drug outcomes or interactions [15,21], including drug–nutrient interactions [22].

Among the latter, in recent years, there has been an increase in interest in drug–nutrient interactions that have an impact on hydration status. In fact, a recently published review provides an overview of the drugs involved in these interactions as well as the potential mechanisms: increase in water elimination through either diarrhoea, urine, or sweat; decrease in thirst or appetite sensation; or central thermoregulation alterations [23]. For example, certain cardiovascular drugs, such as angiotensin-converting enzyme inhibitors or angiotensin II receptor blockers, have a thirst-reducing effect [24,25,26], and diuretics modify urine concentration [24,27,28]. In this context, a pilot study by Puga et al. [29] assessed the effects of chronic use of certain drugs on the hydration status of an elder population and found correlations in patients treated with corticosteroids that varied depending on the route of administration [29]. Moreover, a study by Hoen et al. [30] evaluated the differences in hydration status, analysed by means of bioelectrical impedance, considering patient’s drug treatment and diseases, and observed significant enrichment of certain drugs in either a dehydrated or hyperhydrated patient. However, no associations between altered fluid status and specific medications were identified [30]. In any case, it is important to point out that diagnosing dehydration is a complex challenge because there is no “gold standard” method, so assessing hydration status should be performed holistically, looking at different parameters rather than focusing on a single tool [31]. In fact, urinary parameters can be further affected by unrelated factors such as food, medication, and illness. For example, urine can be discoloured by certain foods and/or drugs, or the presence of blood [6], and urine markers are less useful for determining hydration status in older adults because of their reduced ability to concentrate urine [32].

Despite the importance of maintaining adequate hydration status in the elderly to ensure healthy ageing, and the high prevalence of multiple drug use in this population group, research on the impact of chronic drug consumption on the hydration status is limited. In this context, we hypothesised that the chronic consumption of certain drugs may alter the hydration status. Therefore, the aim of the present study was to assess the hydration status of a sample of older adults in a holistic manner (body composition analysis, urinary biochemical parameters, and water balance) and to investigate the potential associations with chronic drug use according to total drug consumption and Anatomical Therapeutic Chemical (ATC) classification. For example, since certain cardiovascular drugs reduce thirst, we will test for an association between these drugs and lower water intake. In addition, due to diuretics effect on urine concentration, we will test for an association between these drugs and urine biochemical parameters. Finally, we will test the associations between drug consumption and total body water.

## 2. Materials and Methods

### 2.1. Study Protocol

This cross-sectional observational study was carried out from October 2019 to May 2022. According to data provided by National Institute of Statistics from Spain in 2018, 563,654 old adults lived in the Community of Madrid (Spain) [33]. For a proper estimation of the sample size and considering achieving an accuracy of 10.0% in an estimated proportion, using a normal asymptotic 95.0% bilateral confidence interval, and assuming that the expected proportion of dehydration was 30.0% [10,12,13], 81 individuals should have been included in the study. Considering a 10.0% dropout rate, it should have been necessary to recruit approximately 100 volunteers. However, due to COVID-19 pandemic evolution, sample size was adapted to minimise dropout ratio, raising the sample size up to approximately 140 elders.

Volunteer recruitment was carried out in community pharmacies, nursing homes, and the CEU San Pablo University Polyclinic from Madrid, Spain. Given the impact of environmental weather conditions on our aim, the study was limited to the following timeframes of each year: January to May and October to December with average temperatures of 10 °C and 11 °C, respectively, in accordance with the records of the Spanish Meteorological Agency [34].

The inclusion criteria were (a) individuals older than 55 years and (b) individuals mentally and physically healthy. Exclusion criteria were as follows: (a) individuals with renal or water balance-related diseases, suffering from fever, vomiting, or diarrhoea at the time of the study or (b) those with medical devices that prevent body composition analysis, such as pacemakers.

Ethical approval was granted by the Clinical Research Ethics Committee of CEU San Pablo University (ethical code 234/17/06). The study was performed in accordance with the ethical standards laid down in the Declaration of Helsinki and its later amendments. Participants were informed about the objectives of the study and the procedures involved and signed an informed consent prior to their inclusion in the study. All personal data were confidential and only researchers assigned to the project had access to them, complying with the General Data Protection Regulation 2016/679 and the Organic Law 3/2018 for the Protection of Personal Data and the guarantee of digital rights.

**Figure 1:**
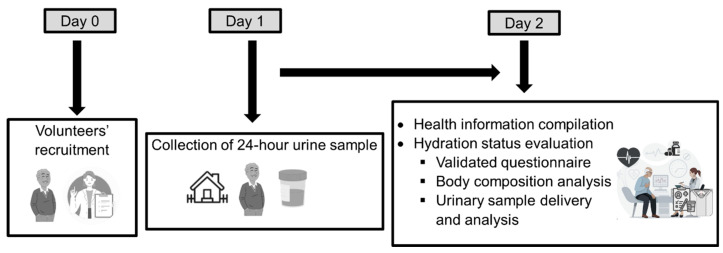
Protocol of the study.

The protocol of the study (Figure 1) was explained to the potential volunteers prior to their inclusion.

### 2.2. Health Information Compilation

Volunteers’ health information was compiled, including drug consumption (over-the-counter, prescription, and/or traditional and complementary medicines) and posology. Only drugs of chronic treatments, i.e., regularly used in the last 6 months, were considered. Drugs were classified into eight groups (cardiovascular, respiratory, gastrointestinal, endocrine, nervous system, genito-urinary, and musculoskeletal) according to the Anatomical Therapeutic Chemical (ATC) classification [35] and to their effect on the maintenance of adequate hydration status (diuretics) [23]. Cardiovascular drugs included antihypertensives (such as angiotensin-converting enzyme inhibitors or angiotensin II receptor antagonists, among others), hypercholesteraemic, anti-coagulants, and antiplatelet agents. Respiratory drugs included corticosteroids and bronchodilators, and drugs for the gastrointestinal tract included antacids, gastric mucosal protectants (such as proton pump inhibitors), and prokinetics. Endocrine group encompassed hypoglycaemic agents and thyroid hormone substitutes, whereas anxiolytics, antidepressants, and antipsychotics were included in the nervous system one. Finally, the genito-urinary group included drugs indicated for the treatment of benign prostatic hyperplasia or overactive bladder, whereas the musculoskeletal one comprised anti-inflammatory and pain management drugs (such as non-steroidal anti-inflammatory drugs, among others).

### 2.3. Hydration Status Evaluation

#### 2.3.1. Validated Questionnaire

Participants completed the previously validated “The Hydration Status Questionnaire” (HSQ) [36] that allowed the estimation of water intake from food and beverages and water elimination from urine, faeces, and sweat. Water balance was calculated as the difference between total water intake and total water elimination.

#### 2.3.2. Body Composition Analysis

In order to standardise the obtained results, all measurements were conducted under fasting conditions, without liquid consumption in the four hours prior to the tests and without any physical exercise the previous day. Anthropometric measurements were performed according to the recommendations of the International Standards for Anthropometric Assessment (ISAK) [37] by level I- and II-accredited anthropometrists. The anthropometric evaluation comprised the measurement of weight by a SECA^TM^ 877 digital scale with an accuracy of 200 g (Seca GmbH & Co. KG., Hamburg, Germany); height, measured to the nearest 0.1 cm using a wall-mounted stadiometer SECA^TM^ (Seca GmbH & Co. KG, Hamburg, Germany); and waist and hip circumferences, measured with a flexible tape Cescorf™ (Cescorf Equipamentos, Porto Alegre, Brasil).

Weight and height data were used to calculate the body mass index (BMI), or Quetelet index, according to the following formula [38]:

BMI = weight (Kg)/height^2^ (m)

Subjects were classified as underweight (BMI < 18.5 kg/m^2^), normal weight (18.5 kg/m^2^ ≥ BMI ≤ 24.9 kg/m^2^), overweight (25.0 kg/m^2^ ≥ BMI ≤ 29.9 kg/m^2^), and obese (BMI ≥ 30 kg/m^2^) according to WHO categorisation [39]. Moreover, the waist-to-hip ratio was calculated as the ratio of the waist circumference (cm) and the hip circumference (cm). Lastly, body composition (body fat, total body water, and phase angle 50 Hz) was estimated by bioelectrical impedance (BIA) with a Bioscan Spectrum Multifrequency^TM^ (Maltron International Ltd., Rayleigh, UK).

#### 2.3.3. Urinary Analysis

During the recruitment, subjects were briefed on how to collect their 24 h urine samples in the urine containers provided using the following procedure: on the morning of day 1, upon waking, participants voided and then discarded this first-morning void. Then, they collected all subsequent voids in the provided urine containers, including voids produced during the night and their first urine void of day 2, thus completing the 24 h collection. Samples were kept under refrigeration (4 °C) until analysis.

Urine colour was determined using a colour scale (range 1–8) developed by Armstrong et al. [40]. Urine-specific gravity and density were measured using an MISCO^TM^ Palm Abbe digital refractometer. Osmolality was determined by an osmometer, sodium and potassium by flame emission photometry, and creatinine by the Jaffé colorimetric method [41]. A urine sample was considered complete if the creatinine level was >0.4 g/24 h for women and >0.6 g/24 h for men [42] or if the volume collected was >500 mL [43].

### 2.4. Statistical Analysis

The statistical analysis was performed using SPSS 29.0 software for Windows (IBM Corp., Armonk, NY, USA). Data were expressed according to the nature of the variable as mean ± standard deviation or median and interquartile range for quantitative variables or in frequencies for qualitative variables. The variables were tested for normality using the non-parametric Kolmogorov–Smirnov test. To compare the difference in medians, the Mann–Whitney *U*-test was used when comparing sex groups. Significant differences were considered when *p* ≤ 0.05. To examine confounding variables, 2 × 2 contingency tables were used for analysis. Fisher’s exact test was applied to determine if there was a significant association between qualitative variables for sexes. Multiple regression analysis was performed to examine the relative contribution of predictor variables (in terms of (i) total number of drugs consumed and (ii) total number of drugs consumed from the different groups analysed) to the effect (parameters indicative of hydration status, i.e., water intake, urinary pH and creatinine, and total body water). Regression models were adjusted for age, sex, and total urine volume.

## 3. Results

Volunteers (*n* = 144) aged 55 to 96 years old were selected and stratified according to their pharmacological treatment. According to their sex, 61 men (42.4%) and 83 women (57.6%) participated in the study, with a median age of 68 and 67 years old, respectively.

In Table 1, volunteers were stratified according to whether they were drug users or not. The most prescribed drugs were represented by cardiovascular drugs (68.1%), gastrointestinal drugs (36.8%), endocrine drugs (32.6%), and drugs for the nervous system (31.3%) (Table 2). Among patients consuming cardiovascular medications, the most frequently prescribed drugs were the cholesterol-lowering drugs atorvastatin and simvastatin (*n* = 34; 23.6% and *n* = 20; 13.9%, respectively), the antiplatelet agent acetylsalicylic acid (*n* = 22; 15.3%) and the antihypertensive drug enalapril (*n* = 15; 10.4%). Moreover, it was also found that women were significantly more likely to consume drugs for the nervous system (*p* ≤ 0.05) with respect to men, while men were significantly more likely to use drugs for genito-urinary disorders then women (*p* ≤ 0.001).

Results from the validated hydration questionnaire are presented in Table 3. As it can be observed, no significant differences were found between sexes concerning the intake of water and water from beverages. However, water intake from food was significantly higher (*p* ≤ 0.05) in men than in women. Oppositely, the intake of water and water from beverages normalised by body weight was significantly higher in women (*p* ≤ 0.05) than in men. Likewise, when water intake was normalised by free fat mass, it was observed that water, water from beverages, and total water intake were significantly higher in women than in men (*p* ≤ 0.001). Furthermore, no significant differences between sexes were determined regarding total water intake, total water elimination, and water balance. However, there was a trend for women to have a lower water balance than men.

Multiple regression models were used to evaluate the cross-sectional associations between drug use and total water intake/body weight. As shown in Table 4, cardiovascular drug consumption, adjusted by age, in men was significantly associated with lower total water intake normalised by body weight (*p* = 0.029). In fact, when comparing the average water intake in men, it was found that total water intake normalised by body weight was significantly lower in men treated with cardiovascular drugs than in those untreated (29.5 mL/Kg vs. 35.4 mL/Kg, *p* = 0.030). Conversely, this association was not found in women. However, in this latter group, a significant positive association was found between musculoskeletal drug consumption, adjusted by age, and total water intake normalised by body weight (*p* = 0.018). No associations were found between total drug consumption, consumption of other drug groups, and water intake neither in men nor in women.

Regarding the characteristics and biochemical parameters in 24 h urine samples of the study participants (Table 5), significant differences between sexes were only found for creatinine concentration, which resulted to be higher in men than in women (*p* ≤ 0.001). All 24 h urine samples were considered complete according to creatine concentration and total urine volume [42,43].

Multiple regression models were used to assess the cross-sectional associations between drug use and urine biochemical parameters (Table 6). Hence, it was observed that total drug consumption, adjusted by age and total urine volume, was significantly associated with lower urine pH (*p* < 0.001) and creatinine (*p* = 0.004) in men. Moreover, also in men, diuretic and endocrine drugs consumption, adjusted by age and total urine volume, was significantly related to low urine pH and creatinine concentration (*p* = 0.004 and *p* = 0.031, and *p* < 0.001 and *p* = 0.026, respectively). Furthermore, cardiovascular, musculoskeletal, and respiratory drugs intake, adjusted by age and total urine volume, was significantly associated with low urine pH (*p* = 0.014, *p* = 0.010, and *p* = 0.024, respectively). However, genito-urinary drug consumption was significantly associated with higher urine pH (*p* = 0.019). Consequently, the greater total drug consumption as well as the chronic use of certain drug groups (diuretics, cardiovascular, endocrine, musculoskeletal, and respiratory drugs) seem to play a remarkable role in the development of negative alterations in the hydration status, whereas genito-urinary drugs lead to the opposite effect. These associations were determined in men, but not in women (Table S1), according to these urine biochemical parameters. Nevertheless, no associations could be observed in the remaining urinary indices.

Anthropometric data are presented in Table 7. As expected, height, weight, waist circumference, and waist-to-hip ratio values were significantly higher in men than in women (*p* ≤ 0.001). However, body fat, both in kg and in %, resulted in higher values in women than in men (*p* ≤ 0.001), revealing higher values of total body water, in kg and in %, in men than in women (*p* ≤ 0.001). However, there were no statistically significant differences between sexes for BMI, hip circumference, and phase angle. BMI results, in both men and women, were indicative of being overweight. 

Likewise, multiple regression models were used to evaluate the cross-sectional associations between drug use and anthropometric parameters (Table 8). Thus, total drug consumption in men, adjusted by age, was significantly associated with lower total body water (*p* = 0.024) In addition, the analysis of the associations between specific drug use and anthropometric parameters revealed that both diuretic and cardiovascular drug consumptions in men, adjusted by age, were significantly associated with lower total body water (*p =* 0.002 and *p =* 0.029, respectively). In fact, total body water in men treated with diuretics and cardiovascular drugs was significantly lower than in those non-treated (46.5% vs. 51.2%, *p* = 0.010 and 49.7% vs. 52.8%, *p* = 0.018). Nevertheless, for genito-urinary drug consumption, adjusted by age, a significant positive association was determined in men for total body water (*p =* 0.017). In the latter, total body water in drug users was significantly higher than in non-users (52.1% vs. 49.6%, *p* = 0.044). However, none of these independent relations were found in women (Table S2). According to these findings, the greater total drug use, as well as the chronic consumption of diuretics, and cardiovascular drugs would negatively affect the hydration status by decreasing the percentage of body water, whereas genito-urinary drugs would positively affect hydration status by increasing the percentage of body water.

## 4. Discussion

The significant increase in the elderly population in recent years, as well as the expected trends, make research into ensuring healthy ageing one of today’s major challenges. In this regard, considering the important role of hydration in health and wellness [44,45], research on the different factors that impact hydration status has gained attention in the last few years, including drug consumption. In fact, our study supports the hypothesis that chronic treatment with certain drugs has an effect on hydration status. However, one of the main limitations to date is the lack of a “gold standard” methodology for its assessment [31], requiring the use multiple approaches that include parameters indicative of water intake as well as biochemical and anthropometric measurements. In our study, a sample of older chronic drug users was selected, and their hydration status was analysed. Results of drug consumption were in line with data from the latest Spanish National Health Survey and the European Health Survey [46].

Regarding water intake, women’s water consumption in our study was in line with the European Food Safety Authority (EFSA) requirements (2.0 L/day), while men’s consumption was very close to these recommendations (2.5 L/day) [41]. These results were much higher than those obtained for the population in this age group in the Spanish representative Anthropometry, Intake, and Energy Balance in Spain (ANIBES) study (1.59 L/day and 1.58 L/day for men and women, respectively) [47] but more similar than those obtained by Malisova et al. [48] who reported in a sample of independent older adults aged 65 to 81 years a total water intake of 2571 ± 739 mL/day. Considering that one of the age-related physiological factors contributing to dehydration is a reduced sense of thirst, owing to the diminished responsiveness of the osmoreceptors and the decrease in angiotensin I levels [49], these results may be interpreted with caution. Moreover, it is important to note that these determinations were made using a subjective methodology that may be influenced by patient’s memory, which may be altered due to their age, as previously reported by Archer et al. [50]. In any case, the prevalence of negative water balance in the study sample was 46.5%, slightly higher than that obtained in previous studies [10,12,13].

When analysing the associations between drug consumption and water intake, we observed that cardiovascular drug consumption in men was significantly associated with lower total water intake normalised by body weight, i.e., men on chronic treatment with these drugs had lower water intake. In fact, total water intake normalised by body weight in men who are users of cardiovascular drugs was significantly lower compared to those non-users. This finding may be related to the fact that some of the drugs in the cardiovascular group are known to have a thirst-reducing effect, such as angiotensin-converting enzyme inhibitors or angiotensin II receptor blockers [23]. To our knowledge, the thirst-reducing effect of these drugs has been mainly studied in animal models [25,26], and research in humans on this issue is scarce. However, a study in France using the pharmacovigilance system to evaluate adverse drug reactions in elderly patients during a heat wave revealed metabolic adverse drug reactions, including dehydration and other hydroelectrolyte disorders, and identified angiotensin-converting enzyme inhibitors and angiotensin II receptor blockers as drugs responsible for these effects [24]. In this regard, it is important to point out that when thirst sensation appears our body is usually already dehydrated [51]. The fact that musculoskeletal drugs are associated with an increase in water intake may be related to their effect on kidney function, which can lead to an increase in thirst, as previously reported [52].

Our holistic evaluation of the hydration status included the analysis of various biochemical parameters in a 24 h urine sample. Significant differences among sexes were only found for creatine concentration. This finding is associated with the lower muscle mass in women in comparison to men, in agreement with several previous findings [53,54,55]. In any case, urine creatinine was used not only to assess hydration status, but also to ensure complete urine collection along with total urine volume [42,43]. Concerning the associations between drug consumption and the urinary biochemical parameters, no associations were found between drug use and specific gravity or osmolality. Note that high values for both osmolality and specific gravity indicate dehydration (osmolality > 766 mOsm/kg and specific gravity > 1.020 g/L) [56]. However, in the elderly, these urinary indices tend to be “falsely” low due to age-related reduced urine concentrating ability [45,57]. In fact, it has been estimated that older people, aged 60–79, have about 20% less maximum urine concentration capacity than younger adults [58], and this may be hindering the establishment of associations with drug use. In this regard, the new guidelines on nutrition and hydration in older people advise against using urine colour or specific gravity to evaluate the hydration status in the elderly [59]. However, interestingly, a trend towards higher specific gravity and osmolality values was observed in treated patients compared with untreated patients. For example, men treated with diuretics had a specific gravity of 1.012 (g/L) vs. 1.015 (g/L) in those untreated, while the osmolality of treated men was 456.50 mOsm/kg vs. 494.50 mOsm/kg in those non-treated.

Conversely, inverse associations between drug use and urinary pH and creatinine levels were determined. In this sense, it is important to point out that low urinary pH, i.e., acidic urines, are indicative of dehydration [60]. Specifically, total drug use, cardiovascular, diuretic, endocrine, musculoskeletal, and respiratory drug use were significantly related to lower pH, whereas genito-urinary drug consumption was significantly associated with higher pH. These differences may be explained because drugs in the genito-urinary group include some used to treat benign prostatic hyperplasia and overactive bladder and hence aim to reduce urine volume [61,62], thereby improving hydration status. Oppositely, total drug consumption and cardiovascular, diuretic, endocrine, musculoskeletal, and respiratory use were associated with worse hydration status. Furthermore, low urinary creatinine concentrations are also related to dehydration [63,64,65]. Therefore, the associations found for diuretics and total drug consumption confirm our initial hypothesis. These results were in line with those of a pilot study that found correlations between urinary dehydration index and diuretic and endocrine chronic consumption [29].

Finally, the effect of drug consumption on hydration status was confirmed in terms of total body water analysed by means of electrical impedance. Hence, according to our findings, total drug consumption, diuretics, and cardiovascular drugs are inversely associated with total body water, whereas genito-urinary drugs have a positive effect. As previously stated, these differences may be attributable to the different mechanisms of action of genito-urinary vs. cardiovascular or diuretic drugs, resulting in opposite effects on body composition [23,61,62]. These results were in line with those of Hoen et al. [30] who found associations between elderly patients’ hydration statuses, measured by bioelectrical impedance, and cardiovascular, dementia, renal, and diuretic diseases. These authors also found that specific drugs such as olmesartan (cardiovascular) or furosemide and torasemide (diuretics) were significantly enriched in the patients analysed [30]. Likewise, a pilot study also showed negative correlations between total body water and diuretic consumption (furosemide, spironolactone, and total diuretics) [29].

Despite interesting findings in men, our study showed an apparent lack of association between drug consumption and hydration status in women. In this sense, and in view of the high degree of novelty of our topic, further studies seem to be necessary in order to identify other factors that may be modulators of this association. In this context, it should be noted that although the women in the study reported a significantly higher intake of water and other beverages than the men, their total body water was significantly lower.

Older adults are more prone to dehydration, especially those living with multiple chronic diseases or on chronic treatment with certain drugs. However, despite the importance of maintaining hydration status in the health and quality of life of older adults, its alterations are under-recognised and poorly addressed. Dehydration has been related to urological, gastrointestinal, circulatory, and neurological alterations [10], the latter including cognitive performance and mood [66], and it is an independent factor of the hospital length of stay, readmission, intensive care, in-hospital mortality, and poor prognosis [45], with the subsequent economic impact for society. In this regard, as shown in our study, there is a real need for further research into drug–nutrient interactions affecting water in order to prevent or control problems associated with dehydration.

### Strengths and Limitations

The main strength of this study is its novelty; as to our knowledge, there has been little research on the associations between drug use and hydration status despite the important health implications. Another strength is the use of a multi-approach to assess hydration status, combining a validated questionnaire and objective methods such as urinalysis or anthropometric assessment by bioelectric impedance.

In terms of limitations, the cross-sectional design of the study prevents the establishment of causal relationships with the other study factors. Moreover, it is important to emphasise the complexity of the studied population, a convenience sample, with multiple pathologies in addition to age-related conditions, which may have prevented the establishment of more associations between drug consumption and hydration status. Likewise, it is important to note that despite total body water from bioimpedance being technically not valid when hydration condition varies, a greatest accuracy is achieved under standardised conditions, for example, cleaning the skin with alcohol wipes before placing electrodes, taking accurate measurements of height and weight, fasting for 4 h prior to the test, and avoiding exercise, as performed in our study.

In this sense, it may be interesting for further studies to evaluate these associations in a younger and representative sample in order to avoid this potential confounding factor and to assess hydration status with a biochemical parameter in blood, such as plasma osmolality, which, according to the most recent studies, seems to be the most reliable marker of dehydration. This would make the holistic study of hydration status even more exhaustive, given the widely acknowledged difficulty of its analysis.

## 5. Conclusions

Results from our study confirmed the effect of chronic treatment with certain drugs on hydration status. Nutritional interventions to prevent complications of altered hydration status, before they become irreversible, may be of great interest in certain populations, especially in highly vulnerable groups such as the elderly.

## Figures and Tables

**Table 1:** Percentage of drugs consumed in the study population.

	Total Population (*n* = 144)	Men (*n* = 61)	Women (*n* = 83)
Non-drug users (%)	11.1	9.8	12.0
Drug users (%)	88.9	90.2	88.0

Data are presented as percentages (%).

**Table 2:** Percentage of drugs consumed according to therapeutic indication in the study population.

	Total Population (*n* = 144)	Men (*n* = 61)	Women (*n* = 83)
Cardiovascular (%)	68.1	73.8	63.9
Gastrointestinal (%)	36.8	42.6	32.5
Endocrine (%)	32.6	32.8	32.5
Nervous (%)	31.3	23	37.3 *
Diuretics (%)	25.7	27.9	24.1
Musculoskeletal (%)	20.1	23.0	18.1
Genito-urinary (%)	15.3	31.1	3.6 ***
Respiratory (%)	9.0	11.5	7.2

Data are presented as percentages. * *p* ≤ 0.05 indicates significant differences between sexes (Fisher’s exact test); *** *p* ≤ 0.001 indicates significant differences between sexes (Fisher’s exact test).

**Table 3:** Water intake, elimination, and balance in the study population.

	Total Population (*n* = 144)	Men (*n* = 61)	Women (*n* = 83)
Water intake (L/day)	1.00 (0.60–1.50)	1.00 (0.60–1.40)	1.00 (0.75–1.50)
Water intake from beverages (L/day)	1.67 (1.27–2.1)	1.68 (1.37–2.01)	1.63 (1.21–2.30)
Water intake from food (L/day)	0.61 (0.43–0.89)	0.69 * (0.48–0.89)	0.50 (0.42–0.89)
Total water intake (L/day)	2.31 (1.82–2.91)	2.31 (2.03–2.90)	2.30 (1.71–2.93)
Total water elimination (L/day)	2.21 (1.89–2.63)	2.15 (1.84–2.54)	2.24 (1.97–2.75)
Water balance (L/day)	0.04 (−0.43–0.54)	0.14 (−0.17–0.72)	−0.08 (−0.61–0.49)
Water intake/body weight (mL/Kg)	13.03 (8.99–19.43)	11.98 * (7.50–16.51)	14.99 (9.77–21.19)
Water intake from beverages/weight (mL/Kg)	23.35 (16.65–30.33)	21.20 * (16.82–24.88)	25.00 (16.26–32.62)
Water intake from food/weight (mL/Kg)	7.78 (6.07–11.89)	8.73 (6.55–11.67)	7.06 (5.59–12.36)
Total water intake/body weight (mL/Kg)	31.45 (24.28–42.11)	30.60 (24.53–36.96)	32.50 (24.23–43.95)
Water intake/free fat mass (mL/Kg)	22.47 (14.96–33.33)	17.85 *** (11.07–25.64)	26.81 (18.82–42.86)
Water intake from beverages/free fat mass (mL/Kg)	37.24 (29.04–53.17)	32.27 *** (26.57–38.53)	48.23 (31.72–63.94)
Water intake from food/free fat mass (mL/Kg)	13.91 (10.39–19.87)	13.49 (9.93–18.40)	14.33 (10.64–24.30)
Total water intake/free fat mass (mL/Kg)	53.85 (41.50–73.45)	45.97 *** (37.83–56.02)	66.87 (45.90–80.88)

Data are presented as median and interquartile range. * *p* ≤ 0.05 indicates significant differences between sexes (Mann–Whitney *U*-test). *** *p* ≤ 0.001 indicates significant differences between sexes (Mann–Whitney *U*-test).

**Table 4:** Cross-sectional associations between drug use and total water intake/body weight.

	Men							
	Non-Adjusted				Adjusted by Age			
**Total drug consumption**								
Variable	*β*	SEM	*p-value*	*r*^2^ adjusted	*β*	SEM	*p-value*	*r*^2^ adjusted
Total water intake/body weight (mL/Kg)	−0.038	0.048	0.773	−0.016	−0.074	0.046	0.557	0.076
**Total cardiovascular drug consumption**								
Variable	*β*	SEM	*p-value*	*r*^2^ adjusted	*β*	SEM	*p-value*	*r*^2^ adjusted
Total water intake/body weight (mL/Kg)	−0.271	0.019	**0.034**	0.058	−0.282	0.020	**0.029**	0.052
**Total gastrointestinal drug consumption**								
Variable	*β*	SEM	*p-value*	*r*^2^ adjusted	*β*	SEM	*p-value*	*r*^2^ adjusted
Total water intake/body weight (mL/Kg)	0.139	0.008	0.284	0.003	0.101	0.008	0.414	0.112
**Total endocrine drug consumption**								
Variable	*β*	SEM	*p-value*	*r*^2^ adjusted	*β*	SEM	*p-value*	*r*^2^ adjusted
Total water intake/body weight (mL/Kg)	−0.195	0.011	0.132	0.022	−0.210	0.011	0.107	0.025
**Total diuretic drug consumption**								
Variable	*β*	SEM	*p-value*	*r*^2^ adjusted	*β*	SEM	*p-value*	*r*^2^ adjusted
Total water intake/body weight (mL/Kg)	−0.105	0.006	0.419	−0.006	−0.112	0.007	0.398	−0.020
**Total musculoskeletal drug consumption**								
Variable	*β*	SEM	*p-value*	*r*^2^ adjusted	*β*	SEM	*p-value*	*r*^2^ adjusted
Total water intake/body weight (mL/Kg)	0.109	0.008	0.403	−0.005	0.074	0.008	0.557	0.082
**Total genito-urinary drug consumption**								
Variable	*β*	SEM	*p-value*	*r*^2^ adjusted	*β*	SEM	*p-value*	*r*^2^ adjusted
Total water intake/body weight (mL/Kg)	0.205	0.009	0.113	0.026	0.165	0.008	0.176	0.145
**Total respiratory drug consumption**								
Variable	*β*	SEM	*p-value*	*r*^2^ adjusted	*β*	SEM	*p-value*	*r*^2^ adjusted
Total water intake/body weight (mL/Kg)	0.110	0.010	0.399	0.005	−0.089	0.015	0.498	−0.014
**Total drug consumption**								
Variable	*β*	SEM	*p-value*	*r*^2^ adjusted	*β*	SEM	*p-value*	*r*^2^ adjusted
Total water intake/body weight (mL/Kg)	0.178	0.129	0.107	0.020	0.184	0.026	0.065	0.209
**Total cardiovascular drug consumption**								
Variable	*β*	SEM	*p-value*	*r*^2^ adjusted	*β*	SEM	*p-value*	*r*^2^ adjusted
Total water intake/body weight (mL/Kg)	0.179	0.011	0.105	0.020	0.183	0.010	0.081	0.119
**Total gastrointestinal drug consumption**								
Variable	*β*	SEM	*p-value*	*r*^2^ adjusted	*β*	SEM	*p-value*	*r*^2^ adjusted
Total water intake/body weight (mL/Kg)	0.023	0.005	0.838	−0.012	0.027	0.004	0.804	0.066
**Total endocrine drug consumption**								
Variable	*β*	SEM	*p-value*	*r*^2^ adjusted	*β*	SEM	*p-value*	*r*^2^ adjusted
Total water intake/body weight (mL/Kg)	−0.039	0.007	0.728	−0.011	−0.038	0.007	0.735	−0.019
**Total diuretic drug consumption**								
Variable	*β*	SEM	*p-value*	*r*^2^ adjusted	*β*	SEM	*p-value*	*r*^2^ adjusted
Total water intake/body weight (mL/Kg)	0.095	0.005	0.392	−0.003	0.098	0.004	0.362	0.046
**Total musculoskeletal drug consumption**								
Variable	*β*	SEM	*p-value*	*r*^2^ adjusted	*β*	SEM	*p-value*	*r*^2^ adjusted
Total water intake/body weight (mL/Kg)	0.245	0.006	**0.** **025**	0.049	0.249	0.006	**0.** **018**	0.135
**Total genito-urinary drug consumption**								
Variable	*β*	SEM	*p-value*	*r*^2^ adjusted	*β*	SEM	*p-value*	*r*^2^ adjusted
Total water intake/body weight (mL/Kg)	0.194	0.002	0.079	0.026	0.195	0.002	0.079	0.020
**Total respiratory drug consumption**								
Variable	*β*	SEM	*p-value*	*r*^2^ adjusted	*β*	SEM	*p-value*	*r*^2^ adjusted
Total water intake/body weight (mL/Kg)	−0.018	0.005	0.870	−0.012	−0.016	0.005	0.886	0.016

SEM: Standard Error of the Mean.

**Table 5:** Urine biochemical parameters in the study population.

	Total Population (*n* = 144)	Men (*n* = 61)	Women (*n* = 83)
Total urine volume (L/24 h)	1.6 (1.2–2.0)	1.6 (1.2–2.0)	1.7 (1.2–1.9)
Urine colour	4.0 (3.0–6.0)	4.0 (3.0–6.0)	3.0 (3.0–6.0)
pH	6.1 (5.7–6.5)	6.0 (5.7–6.5)	6.1 (5.7–6.5)
Specific gravity (g/L)	1.013 (1.010–1.016)	1.013 (1.011–1.017)	1.012 (1.001–1.016)
Sodium (mEq/24 h)	1855.1 (1289.7–2642.9)	1951.4 (1289.7–2704.3)	1805.8 (1280.7–2402.5)
Potassium (mEq/24 h)	1282.1 (975.4–1709.5)	1312.7 (1067.2–1823.6)	1273.0 (935.7–1679.7)
Sodium/potassium ratio	1.44 (1.09–1.87)	1.30 (1.07–1.87)	1.52 (1.11–1.87)
Osmolality (mOsm/kg)	433.5 (333.0–548.0)	471.0 (340.5–590.3)	422.0 (319.5–520.3)
Creatinine (mg/24 h)	829.8 (570.5–1378.1)	1128.3 *** (724.2–1757.7)	732.8 (542.7–1049.0)

Data are presented as median and interquartile range. *** *p* ≤ 0.001 indicates significant differences between sexes (Mann–Whitney *U*-test).

**Table 6:** Cross-sectional associations between drug use and urinary parameters in men.

	Non-Adjusted				Adjusted by Age and Total Urine Volume			
**Total drug consumption**								
Variable	*β*	SEM	*p-value*	*r*^2^ adjusted	*β*	SEM	*p-value*	*r*^2^ adjusted
pH	−0.381	0.727	**0.001**	0.260	−0.422	0.721	**<0.001**	0.310
Creatinine (mg/24 h)	−0.375	0.001	**0.001**		−0.373	0.001	**0.004**	
**Total cardiovascular drug consumption**								
Variable	*β*	SEM	*p-value*	*r*^2^ adjusted	*β*	SEM	*p-value*	*r*^2^ adjusted
pH	−0.291	0.339	**0.021**	0.111	−0.316	0.348	**0.014**	0.118
Creatinine (mg/24 h)	−0.238	0.000	0.057		−0.213	0.000	0.132	
**Total gastrointestinal drug consumption**								
Variable	*β*	SEM	*p-value*	*r*^2^ adjusted	*β*	SEM	*p-value*	*r*^2^ adjusted
pH	−0.093	0.131	0.445	0.144	−0.098	0.127	0.425	0.163
Creatinine (mg/24 h)	−0.405	0.000	**0.001**		−0.360	0.000	**0.010**	
**Total endocrine drug consumption**								
Variable	*β*	SEM	*p-value*	*r*^2^ adjusted	*β*	SEM	*p-value*	*r*^2^ adjusted
pH	−0.414	0.188	**<0.001**	0.161	−0.482	0.182	**<0.001**	0.260
Creatinine (mg/24 h)	−0.136	0.000	0.259		−0.291	0.000	**0.026**	
**Total diuretic drug consumption**								
Variable	*β*	SEM	*p-value*	*r*^2^ adjusted	*β*	SEM	*p-value*	*r*^2^ adjusted
pH	−0.333	0.108	**0.008**	0.126	−0.372	0.110	**0.004**	0.138
Creatinine (mg/24 h)	−0.213	0.000	0.086		−0.305	0.000	**0.031**	
**Total musculoskeletal drug consumption**								
Variable	*β*	SEM	*p-value*	*r*^2^ adjusted	*β*	SEM	*p-value*	*r*^2^ adjusted
pH	−0.287	0.132	**0.023**	0.115	−0.323	0.130	**0.010**	0.176
Creatinine (mg/24 h)	−0.250	0.000	**0.046**		−0.238	0.000	0.083	
**Total genito-urinary drug consumption**								
Variable	*β*	SEM	*p-value*	*r*^2^ adjusted	*β*	SEM	*p-value*	*r*^2^ adjusted
pH	0.345	0.149	**0.007**	0.095	0.296	0.148	**0.019**	0.173
Creatinine (mg/24 h)	−0.083	0.000	0.507		−0.077	0.000	0.577	
**Total respiratory drug consumption**								
Variable	*β*	SEM	*p-value*	*r*^2^ adjusted	*β*	SEM	*p-value*	*r*^2^ adjusted
pH	−0.297	0.167	**0.021**	0.070	−0.300	0.174	**0.024**	0.055
Creatinine (mg/24 h)	−0.115	0.000	0.393		−0.114	0.000	0.433	

SEM: Standard Error of the Mean.

**Table 7:** Anthropometric characteristics of the population studied according to sex.

	Men (*n* = 61)	Women (*n* = 83)
Weight (kg)	78.4 *** (70.8–85.3)	68.5 (62.4–75.9)
Height (m)	1.68 *** (1.65–1.73)	1.58 (1.53–1.61)
Body mass index (kg/m^2^)	27.4 (24.9–29.4)	27.5 (25.0–31.2)
Waist circumference (cm)	100.0 *** (94.0–105.8)	94.0 (85.8–100.9)
Hip circumference (cm)	104.0 (100.2–108.0)	106.0 (100.0–112.0)
Waist-to-hip ratio	0.97 *** (0.93–1.01)	0.89 (0.83–0.93)
Body fat (kg)	25.4 *** (22.5–30.5)	33.2 (27.6–37.7)
Body fat (%)	33.8 *** (30.3–36.8)	47.5 (43.5–50.7)
Total body water (L)	39.6 *** (35.9–42.1)	29.9 (27.9–31.5)
Total body water (%)	50.1 *** (46.6–54.0)	43.1 (40.4–46.0)

Data are presented as median and interquartile range. *** *p* ≤ 0.001 indicates significant differences between sexes (Mann–Whitney *U*-test).

**Table 8:** Cross-sectional associations between drug use and total body water (%) in men.

	Non-Adjusted				Adjusted by Age			
**Total drug consumption**								
Variable	*β*	SEM	*p-value*	*r*^2^ adjusted	*β*	SEM	*p-value*	*r*^2^ adjusted
Total body water (%)	−0.123	0.095	0.348	−0.002	−0.281	0.089	**0.024**	0.217
**Total cardiovascular drug consumption**								
Variable	*β*	SEM	*p-value*	*r*^2^ adjusted	*β*	SEM	*p-value*	*r*^2^ adjusted
Total body water (%)	−0.228	0.040	0.080	0.036	−0.297	0.042	**0.029**	0.064
**Total gastrointestinal drug consumption**								
Variable	*β*	SEM	*p-value*	*r*^2^ adjusted	*β*	SEM	*p-value*	*r*^2^ adjusted
Total body water (%)	0.129	0.017	0.324	0.000	−0.004	0.016	0.974	0.152
**Total endocrine drug consumption**								
Variable	*β*	SEM	*p-value*	*r*^2^ adjusted	*β*	SEM	*p-value*	*r*^2^ adjusted
Total body water (%)	−0.115	0.022	0.380	−0.004	−0.202	0.022	0.137	0.050
**Total diuretic drug consumption**								
Variable	*β*	SEM	*p-value*	*r*^2^ adjusted	*β*	SEM	*p-value*	*r*^2^ adjusted
Total body water (%)	−0.332	0.012	**0.010**	0.095	−0.408	0.013	**0.002**	0.134
**Total musculoskeletal drug consumption**								
Variable	*β*	SEM	*p-value*	*r*^2^ adjusted	*β*	SEM	*p-value*	*r*^2^ adjusted
Total body water (%)	−0.036	0.017	0.783	−0.016	−0.164	0.017	0.208	0.121
**Total genito-urinary drug consumption**								
Variable	*β*	SEM	*p-value*	*r*^2^ adjusted	*β*	SEM	*p-value*	*r*^2^ adjusted
Total body water (%)	0.395	0.018	**0.002**	0.142	0.298	0.018	**0.017**	0.217
**Total respiratory drug consumption**								
Variable	*β*	SEM	*p-value*	*r*^2^ adjusted	*β*	SEM	*p-value*	*r*^2^ adjusted
Total body water (%)	−0.207	0.021	0.113	0.026	−0.201	0.022	0.147	0.010

## Data Availability

The data presented in this study are available upon reasonable request from the corresponding author. The data are not publicly available due to ethical reasons.

## Supplementary Materials

The following supporting information can be downloaded at: https://www.mdpi.com/article/10.3390/nu16162632/s1, Table S1. Cross-sectional associations between drug use and urinary parameters in women. Table S2. Cross-sectional associations between drug use and total body water (%) in women.
