# Supplementary material for: Drug Consumption and Hydration Status: Analysis of the Associations in an Elder Population
Source: Nutrients. 2024 Aug 9;16(16):2632. doi: 10.3390/nu16162632 (PMC11357581; doi:10.3390/nu16162632)
Supplement: Supplementary file 1 [file nutrients-16-02632-s001.zip › nutrients-3124892-supplementary.pdf]

Table S1. Cross-sectional associations between drug use and urinary parameters in women.

	Non-adjusted				Adjusted by age and total urine volume			
**Total drug consumption**								
**Variable**	***β***	**SEM**	***p-value***	**r^2 adjusted**	***β***	**SEM**	***p-value***	**r^2 adjusted**
pH	0.019	0.541	0.872	-0.017	0.070	0.502	0.513	0.140
Creatinine (mg/24 h)	-0.094	0.001	0.412		-0.053	0.001	0.660	
**Total cardiovascular drug consumption**								
**Variable**	***β***	**SEM**	***p-value***	**r^2 adjusted**	***β***	**SEM**	***p-value***	**r^2 adjusted**
pH	-0.038	0.202	0.744	-0.024	0.006	0.194	0.954	0.071
Creatinine (mg/24 h)	0.032	0.000	0.778		0.027	0.000	0.826	
**Total gastrointestinal drug consumption**								
**Variable**	***β***	**SEM**	***p-value***	**r^2 adjusted**	***β***	**SEM**	***p-value***	**r^2 adjusted**
pH	-0.041	0.086	0.720	-0.024	-0.012	0.085	0.917	0.032
Creatinine (mg/24 h)	-0.013	0.000	0.910		0.057	0.00	0.652	
**Total endocrine drug consumption**								
**Variable**	***β***	**SEM**	***p-value***	**r^2 adjusted**	***β***	**SEM**	***p-value***	**r^2 adjusted**
pH	-0.114	0.120	0.320	-0.004	-0.111	0.122	0.342	-0.028
Creatinine (mg/24 h)	-0.091	0.000	0.425		-0.118	0.000	0.370	
**Total diuretic drug consumption**								
**Variable**	***β***	**SEM**	***p-value***	**r^2 adjusted**	***β***	**SEM**	***p-value***	**r^2 adjusted**
pH	-0.008	0.081	0.943	-0.025	0.023	0.080	0.837	0.011
Creatinine (mg/24 h)	0.040	0.000	0.726		0.007	0.000	0.956	
**Total musculoskeletal drug consumption**								
**Variable**	***β***	**SEM**	***p-value***	**r^2 adjusted**	***β***	**SEM**	***p-value***	**r^2 adjusted**
pH	-0.050	0.117	0.664	-0.023	-0.020	0.113	0.857	0.054
Creatinine (mg/24 h)	0.036	0.000	0.752		0.132	0.000	0.297	
**Total genito-urinary drug consumption**								
**Variable**	***β***	**SEM**	***p-value***	**r^2 adjusted**	***β***	**SEM**	***p-value***	**r^2 adjusted**
pH	0.032	0.031	0.779	0.000	0.048	0.031	0.677	-0.010
Creatinine (mg/24 h)	0.156	0.000	0.171		0.110	0.000	0.271	

Total respiratory drug consumption								
**Variable**	***β***	**SEM**	***p-value***	***r*² adjusted**	***β***	**SEM**	***p-value***	***r*² adjusted**
pH	0.125	0.096	0.266	0.026	0.146	0.096	0.195	0.040
Creatinine (mg/24 h)	-0.194	0.000	0.087		-0.147	0.000	0.247	

SEM: Standard Error of the Mean.

Table S2. Cross-sectional associations between drug use and total body water (%) in women.

	Women							
	**Non-adjusted**				**Adjusted by age**			
**Total drug consumption**								
**Variable**	***β***	**SEM**	***p-value***	***r*² adjusted**	***β***	**SEM**	***p-value***	***r*² adjusted**
Total body water (%)	-0.068	0.086	0.549	-0.008	-0.107	0.077	0.292	0.205
**Total cardiovascular drug consumption**								
**Variable**	***β***	**SEM**	***p-value***	***r*² adjusted**	***β***	**SEM**	***p-value***	***r*² adjusted**
Total body water (%)	-0.009	0.032	0.935	-0.013	-0.038	0.030	0.722	0.099
**Total gastrointestinal drug consumption**								
**Variable**	***β***	**SEM**	***p-value***	***r*² adjusted**	***β***	**SEM**	***p-value***	***r*² adjusted**
Total body water (%)	-0.133	0.014	0.240	0.005	-0.163	0.013	0.126	0.127
**Total endocrine drug consumption**								
**Variable**	***β***	**SEM**	***p-value***	***r*² adjusted**	***β***	**SEM**	***p-value***	***r*² adjusted**
Total body water (%)	-0.176	0.019	0.118	0.019	-0.183	0.019	0.106	0.013
**Total diuretic drug consumption**								
**Variable**	***β***	**SEM**	***p-value***	***r*² adjusted**	***β***	**SEM**	***p-value***	***r*² adjusted**
Total body water (%)	0.073	0.013	0.518	-0.007	0.057	0.013	0.610	0.018
**Total musculoskeletal drug consumption**								
**Variable**	***β***	**SEM**	***p-value***	***r*² adjusted**	***β***	**SEM**	***p-value***	***r*² adjusted**
Total body water (%)	-0.078	0.018	0.491	-0.007	-0.108	0.017	0.316	0.109
**Total genito-urinary drug consumption**								
**Variable**	***β***	**SEM**	***p-value***	***r*² adjusted**	***β***	**SEM**	***p-value***	***r*² adjusted**
Total body water (%)	-0.083	0.005	0.466	-0.006	-0.091	0.005	0.425	-0.009

**Total respiratory drug consumption**								
**Variable**	***β***	**SEM**	***p-value***	***r*² adjusted**	***β***	**SEM**	***p-value***	***r*² adjusted**
Total body water (%)	-0.061	0.015	0.588	-0.009	-0.074	0.015	0.515	0.001

SEM: Standard Error of the Mean.
